# Deep learning-based prediction of rheumatoid arthritis-associated deformity on MRI

**DOI:** 10.1016/j.bas.2025.104328

**Published:** 2025-07-12

**Authors:** Anna Baukje Lebouille-Veldman, Alexander G. Yearley, Timothy R. Smith, Aakanksha Rana, Carmen L.A. Vleggeert-Lankamp

**Affiliations:** a Computational Neuroscience Outcomes Center (CNOC), Department of Neurosurgery, Brigham and Women's Hospital, Harvard Medical School, Boston, MA, USA; b Department of Neurosurgery, Leiden University Medical Center, Leiden, the Netherlands; c Johnson & Johnson Inc, USA

**Keywords:** Rheumatoid arthritis, Deep learning, Artificial intelligence, Prediction model, Cervical spine deformity, Atlantoaxial subluxation, Subaxial subluxation

## Abstract

**Introduction:**

While the prevalence of surgery to correct atlantoaxial subluxation (AAS), subaxial subluxation (SAS) and vertical translocation (VT) in patients with rheumatoid arthritis (RA) had declined, cervical deformity is still observed regularly.

**Research question:**

The objective of this study is to develop a deep learning-based algorithm to predict RA-associated upper cervical spine deformity in patients before or close to RA diagnosis, with the purpose of early risk stratification.

**Materials and methods:**

Patients with RA in which follow-up cervical MRI studies (at least 3 years apart) were available were identified retrospectively in two tertiary care centers. Patients without definitive deformity at baseline were included in the algorithm. Patients were assessed for RA-associated cervical spine deformity, defined as presence of pannus and/or degeneration of the facet joints of C0-C1 and/or C1-C2 on follow up MRI.

**Results:**

Of 3248 patients identified, 220 patients were included in this study, of whom 33 patients developed cervical spine deformity. 153 patients were included for training and sixty-seven for validation of the deep learning-based prediction model. The accuracy of the model was 0.84, with a positive predictive value of 0.56 and a negative predictive value of 0.92.

**Discussion and conclusion:**

A deep learning model was developed to predict the development of pannus and/or facet joint deformity at the craniocervical junction of patients with RA. Future research should focus on large-scale validation of this model with diverse sites and identifying the role of the subaxial spine in the risk of deformity at the level of the craniocervical junction during the course of disease.

## Highlights

• A deep learning algorithm was developed to predict the development of deformity at the craniocervical junction in RA patients.
• The model's accuracy is 0.84 with a positive predictive value of 0.56 and a negative predictive value of 0.92.
• Future research should focus on large-scale validation of this model with diverse sites.

## List of abbreviations

RA =: rheumatoid arthritis
AAS =: atlantoaxial subluxation
SAS =: subaxial subluxation
VT =: vertical translocation
MRI =: magnetic resonance imaging
FU =: follow-up

## 1 Introduction

Rheumatoid arthritis (RA) is a chronic auto-inflammatory disease and commonly defined by inflammatory arthritis of the smaller joints of the hands and feet, but the cervical spine can also be affected (McInnes and Schett, 2011). RA can affect the intricate network of ligaments in the upper cervical spine and cause laxity, which in turn can lead to subluxation of the C1 on C2 vertebrae causing atlantoaxial subluxation (AAS) (Shlobin and Dahdaleh, 2021). ‘Vertical translocation’ (VT) is a severe form of subluxation of the atlantoaxial joint, which is usually accompanied by erosion of the odontoid peg (Kauppi et al., 2005). VT can lead to basilar invagination and cranial settling (Donnally and Varacallo, 2023). Subsequent compression of the spinal cord and medulla oblongata can cause severe neurological deficits and even sudden death (Kauppi et al., 2005).

While the prevalence of cervical involvement in RA declines, it is still observed in patients, even with well-managed disease (Lebouille-Veldman et al., 2023). It was even suggested that up to 86 % of patients with RA have some degree of cervical spine involvement (AAS, SAS and/or VT) (Joaquim and Appenzeller, 2014). Given the severe complications associated with AAS, early diagnosis and treatment are of vital importance in patients with RA (Gillick et al., 2015; Baek et al., 2021; Kotecki et al., 2021). Several studies have attempted to identify risk factors for development and progression of cervical spine deformity in RA (Baek et al., 2021; Inoue et al., 2022; Veldman et al., 2022; Ahn et al., 2011; Ben Tekaya et al., 2023; Bettaieb et al., 2023) since it is crucial to be able to identify these patients early, as this could prevent severe and potentially lethal complications (Gillick et al., 2015). However, no general risk factors have been identified for all RA patients and this prevents clinicians from identifying patients who will eventually develop cervical spine deformity early in their course of disease.

Deep learning has revolutionized survival prediction tasks, achieving unparalleled performance (Goedmakers et al., 2021). In contrast to traditional methods that rely on predefined features and classifiers, these advanced algorithms adopt a unified hierarchical framework for learning intricate visual patterns from imaging data. This hierarchy is constructed through the deployment of a series of cascaded convolutional layers, elevating input images into a multi-dimensional feature space.

While risk stratification models have been developed for the prediction of RA development, no model exists to predict deformity of the upper cervical spine (Momtazmanesh et al., 2022). Early risk stratification with the use of deep learning in this patient population would help clinicians identify patients in need of screening or even early treatment, before they develop severe cervical spine deformity and corresponding complications. The objective of this study is to develop a deep learning-based algorithm to predict RA-associated cervical spine deformity in the upper cervical spine of patients before or close to RA diagnosis.

## 2 Methods

### 2.1 Patient selection

Patients who had multiple magnetic resonance imaging (MRI) scans of their cervical spine at multiple points of follow up (FU) available were identified retrospectively from two tertiary care hospitals. Using RStudio, patients with multiple MRIs of the cervical spine at least 3 years apart were selected (RStudio Team, 2020). Using ICD9 and ICD10 codes for RA (ICD9:714.0, ICD9:714.2, ICD10:M05, ICD10:M05.0, ICD10:M05.1, ICD10:M05.2, ICD10:M05.3, ICD10:M05.4, ICD10:M05.5, ICD10:M05.6, ICD10:M05.7, ICD10:M05.8, ICD10:M05.9, ICD10:M06, ICD10:M06.0, ICD10:M06.8, ICD10:M06.9), it was confirmed whether or not patients were diagnosed with RA. (https://www.aapc.com/codes/icd-10-codes/M06.9.) The diagnosis of RA was also retrospectively verified using digital patient records. Inclusion criteria were diagnosis of RA, availability of at least 2 cervical spine MRIs with at least 3 years of FU between these MRIs, and absence of RA-associated cervical spine deformity on baseline. The study protocol was approved under IRB number 2015P002352. The requirement for informed consent was waived. This research was performed in accordance with the Declaration of Helsinki.

### 2.2 Ground truth

Only the upper cervical spine was considered in the ground truth of this model, so the presence of clinical symptoms of myelopathy and/or the presence of subaxial subluxation were not considered determinants of the status of a patient. This scoring method was validated by a board-certified neurosurgeon. Both baseline and follow-up MRIs were evaluated for each patient. Ratings were independently performed by two experienced observers, whose observations were combined in close collaboration and who were blinded for disease severity of the patient. Observers were trained by a board-certified neurosurgeon to assess MRIs for RA-associated cervical spine deformity. In the case of a discrepancy between the two observers, imaging was reviewed together by both observers and a consensus was reached. If no consensus was reached, conflicts were resolved by a board-certified neurosurgeon, who was also blinded to the disease severity of the patient and the initial scores given by primary observers.

**Fig. 1:**
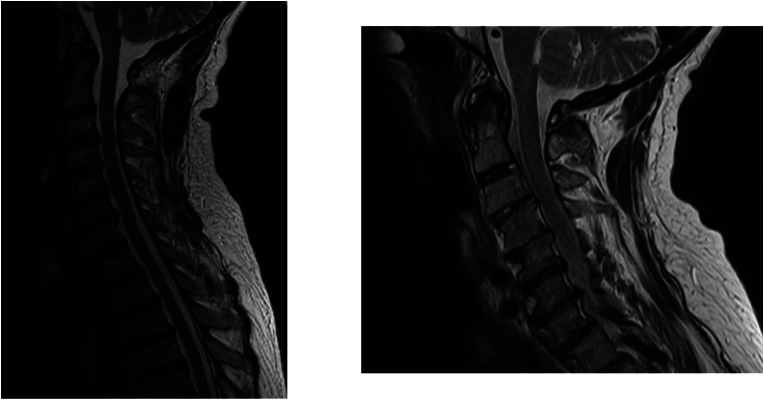
Patient who progressed into a case during FU. On the FU image (right) pannus can be observed, as well as dens erosions. These sagittal T2-weighted MRI images show the progression of a patient from a clean baseline scan to pannus formation and dens erosions on the follow-up scan.

**Fig. 2:**
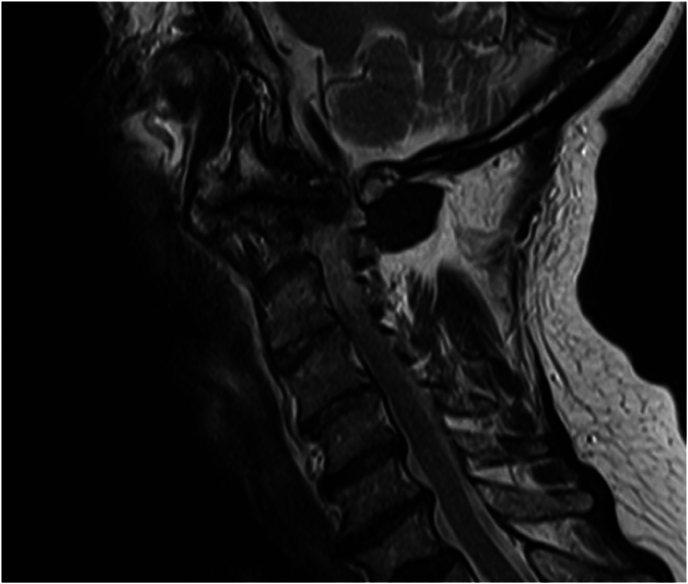
T2 MRI sagittal cervical spine where blossoming of the facet joint can be observed. T2 MRI sagittal cervical spine where blossoming of the facet joint can be observed.

To be marked as a case of RA-associated cervical spine deformity in the model, the follow up MRI had to display either vertical translocation above the line of McRae, visible pannus on T2 MRI, severe dense erosion and/or blossoming of the C0-C1 and/or C1-C2 facet joint(s) (Shlobin and Dahdaleh, 2021; Joaquim et al., 2015; Narvaez et al., 2008; Olah et al., 2020) (Fig. 1, Fig. 2). If only slight hyperintensity between clivus and dens was observed or there was only minimal/doubtful dense erosion, a patient was marked as an intermediate category.

## 3 Data analysis

### 3.1 Model design and training

In this study, we adapted a state-of-the-art 101-layer deep convolutional neural network (CNN) architecture known as Resnet-101, incorporating convolutional layers along with max pooling and average pooling layers. The architectural design retained shortcut connections among every three layers to facilitate the propagation of residual information. Due to its simplicity and success in previous studies, a Resnet architecture was used for the current study (Goedmakers et al., 2021). Our focus was the training of this CNN architecture using T2 sagittal MRIs.

A dataset of total 220 patients were used for training and validation of the model. The dataset was divided into distinct training and test sets following a random sampling strategy, adhering to a partition ratio of 60:40 leading to 153 in training and sixty-seven cases in the test set. During the training process, we implemented on-the-fly data augmentation techniques including center-cropping, flipping, random image brightness/contrast for the training and enhancing the robustness of the model (Buslaev et al., 2020).

For input preprocessing, we selected six middle slices from each (resampled to [1mmx1mmx1mm]) T2 sagittal MRI volume, resulting in an input size of [512x512x6] for the adapted CNN architecture. This standardized input configuration allowed us to effectively evaluate the performance of our model on our prediction task.

The training process utilized a NVIDIA Titan-X graphics processing unit with a 12 GB capacity, operating on an Intel Xeon E7 core i7 machine boasting 64 GB of RAM. The training duration approximated around 20 h. PyTorch, a deep learning library, was employed for the implementation of the proposed models. Our model underwent training with a batch-size of four and initialized with random weights, The training employed a binary cross-entropy loss function, while optimization was carried out using the Adam optimization technique, setting the learning rates at 10^(−4) for 100 epochs (Diederik and Kingma, 2017). Notably, both control parameters, beta and gamma, were consistently set to 10.The Receiver Operator Characteristic (ROC) curve, confusion matrix and relevant testing characteristics (including sensitivity, specificity) has been used as an evaluation metric for validation of this binary classification problem.

### 3.2 Saliency maps

GradCAM saliency maps estimation algorithm has been utilized to highlight important regions in the MR scan for our model's decision, aiding in understanding its predictions by emphasizing key visual cues (Selvaraju et al., 2017).

## 4 Results

### 4.1 Patient characteristics

**Fig. 3:**
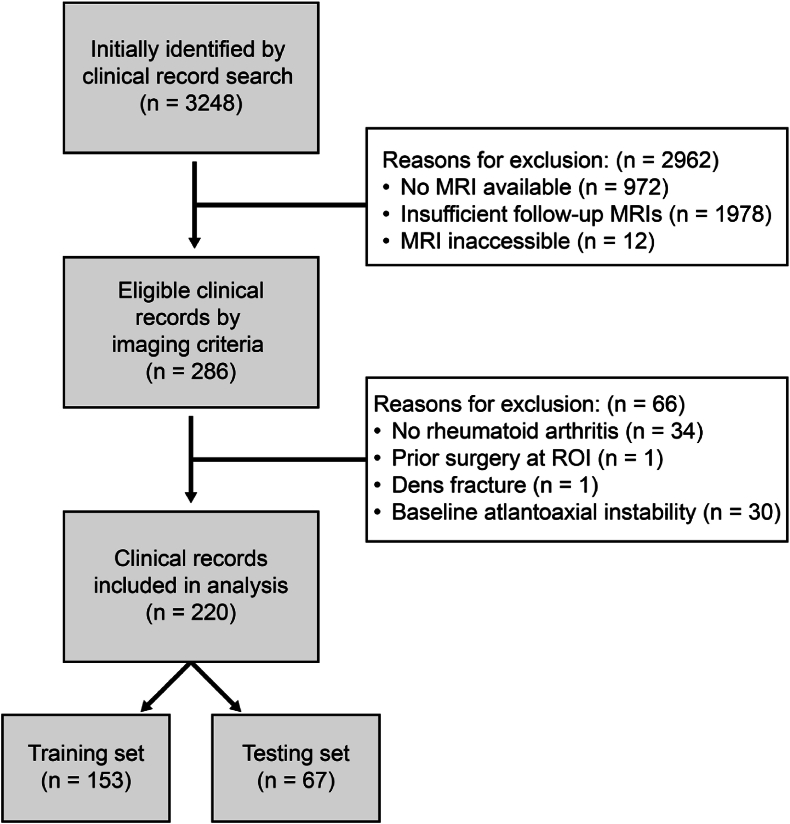
Process of patient inclusion Process of patient inclusion from initially identified patients to the split into training and testing set for the deep learning-based prediction model.

From the retrospective database search, 3248 patients were identified. Of these patients, 2276 had at least one MRI. After applying a filter to select patients with multiple MRIs with at least 1000 days in between, 303 patients met study inclusion criteria. Using ICD codes, the diagnosis of RA was confirmed, which was present for all 303 patients (Fig. 3). Twelve patients were excluded due to the lack of a T2 weighted sagittal cervical spine MRI available at baseline.

During data collection and observation of the ground truth for these 303 patients, fifty-six more patients were excluded. Once the diagnosis RA was manually confirmed by chart review, thirty-four patients were found to have wrongfully received the ICD code for RA. Seven patients did not have an adequate FU scan, one patient had surgery on the craniocervical junction, one patient had an odontoid process fracture.

After determining the ground truth, twenty-nine patients were excluded from the model as they had (severe) cervical spine deformity at baseline already. Therefore, 220 patients were included in the final model. The process of patient inclusion is shown in Fig. 3.

Of the 220 patients included in this study, thirty-three were considered as patients who developed cervical spine deformity in the model. These patients developed definite RA-associated cervical spine deformity from a clean baseline (no RA-associated cervical spine deformity or doubtful RA-associated cervical deformity).

The mean age of the patients in the model was 52 years (SD 13.8) and 188 (86 %) were women. The median follow-up duration between the baseline and follow-up MRI was 7 years (IQR: 4–11) and the median duration of disease at baseline MRI was 2 years (-4-10). The median follow-up duration between baseline and follow-up in the group of patients with cervical deformity after FU was 11 years (IQR 4–14 years), compared to 7 years (IQR 4–10 years) for the patients without cervical spine deformity after FU (Mann-Whitney *U* test, p = 0.13). Demographic characteristics are provided in Table 1.

**Table 1:** Demographics.

Baseline characteristic	Value
Sex, n. females (%)	188 (86 %)
Age at RA diagnosis in years, mean ± SD	52 ± 13.8
Duration of disease in years at baseline MRI, median (IQR)	2 (-4-10)
RF positivity, n. (%)	97 (44 %)
Anti-CCP positivity, n. (%)	64 (29 %)
infliximab used, n. (%)	40 (18 %)
Other biological used, n. (%)	147 (67 %)
Never used biological DMARD, n. (%)	66 (30 %)
FU duration in years, median (IQR)	7 (4–11)

SD = Standard Deviation, MRI = magnetic resonance imaging, IQR = interquartile range, RF = rheumatoid factor, anti-CCP = Anti-cyclic citrulline peptide, n. = number, DMARD = disease modifying anti-rheumatic drug, FU = follow-up.

**Table 2:** Testing characteristics.

Characteristic	Resnet101
Positive	12
Negative	55
True Positive (TP)	9
True Negative (TN)	48
False Positive (FP)	7
False Negative (FN)	3
Sensitivity	0.75
Specificity	0.87
Accuracy	0.84
F1 score	0.75
Positive predictive value (PPV)	0.56
Negative predictive value (NPV)	0.92

### 4.2 Model performance

**Fig. 4:**
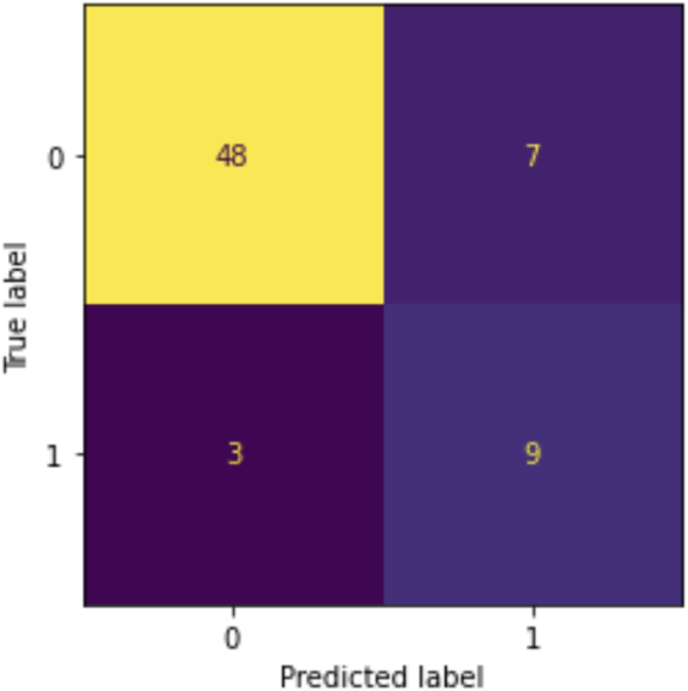
Confusion Matrix Confusion Matrix for the predictions of the algorithm. The x-axis shows the predicted labels, and the y-axis shows the true label (based on the ground truth provided to the model). The discordant predictions and true labels are the falsely predicted cases.

**Fig. 5:**
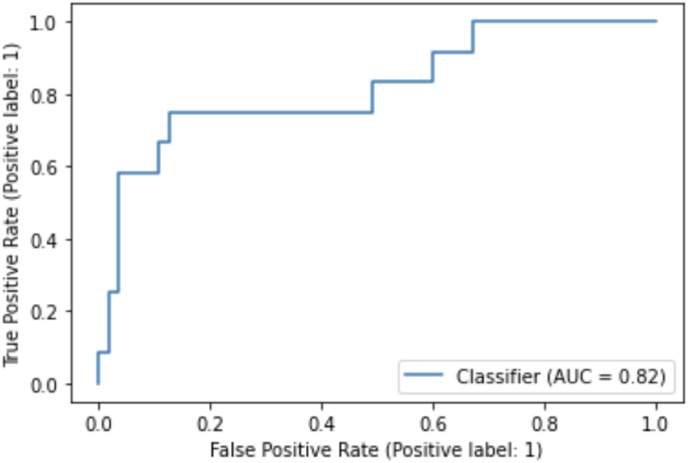
Receiver Operating Characteristic (ROC) curve Receiver Operating Characteristic (ROC) curve of the deep learning-based prediction algorithm.

**Fig. 6:**
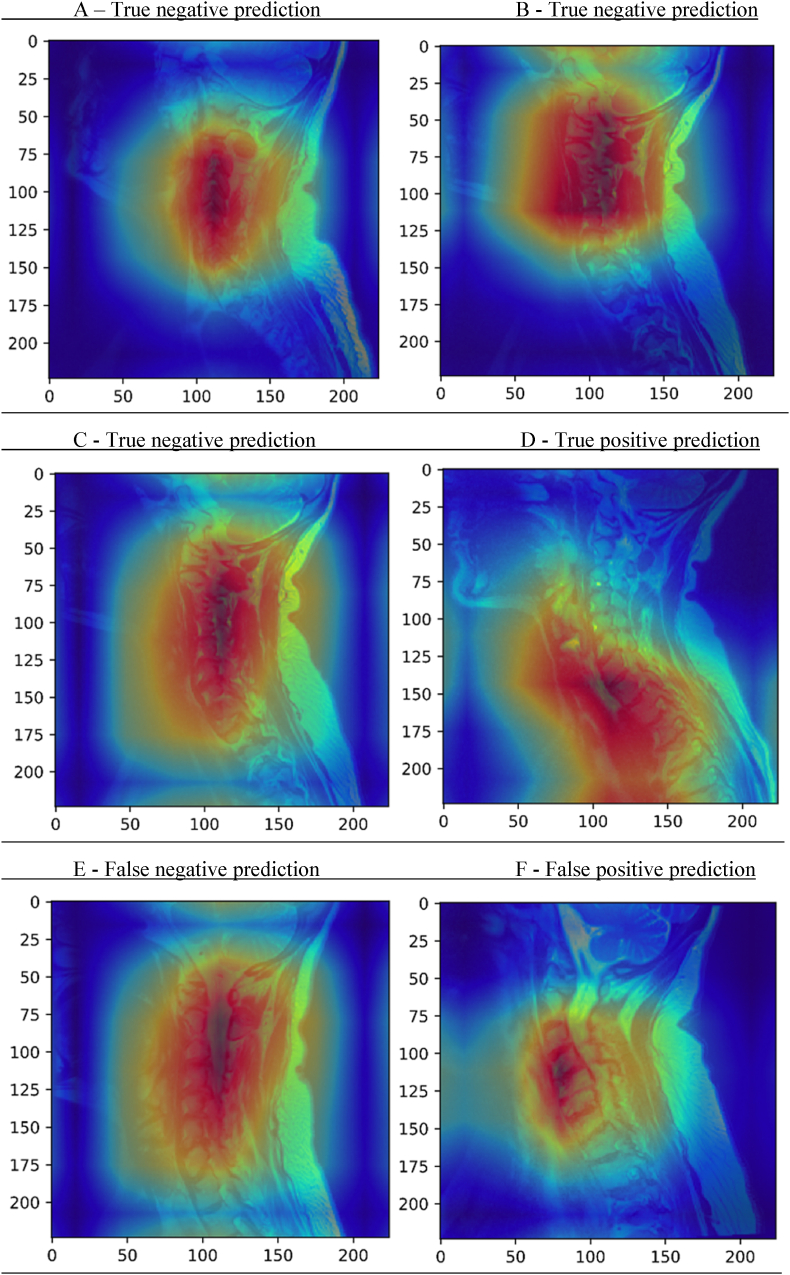
Saliency Maps This figure shows six exemplary Saliency Maps derived from the predictions done by the algorithm. A more intensely red section of the image indicates an area which is more significant for the prediction made by the algorithm. Blue areas indicate areas of less interest. Figures A, B and C show true negative predictions, where the model correctly predicted that the patient would not develop RA-associated cervical spine deformity during the course of disease. Figure D shows a correct prediction that a patient would develop cervical spine deformity and figure E and F show incorrect predictions made by the model.

Results of our deep learning model were showcased in a confusion matrix (Fig. 4) and a Receiver Operating Characteristic (ROC) curve, which showed an area under the curve of 0.82 (Fig. 5). As can be observed in the confusion matrix, the predicted label was correct in 57 of 67 images in the testing set (see Table 2). The model's accuracy is 0.84 with a positive predictive value of 0.56 and a negative predictive value of 0.92. In three patients the model predicted a false negative outcome (Fig. 4). Also, a false positive was predicted in seven cases. To further analyze the performance of the model and explore its predictions, saliency maps were studied. In some of the cases where the model correctly predicted that the patient would not develop cervical spine deformity, focus seemed to be on degeneration of the subaxial cervical spine (Fig. 6A–C), which was also the case for the correctly predicted case who would develop cervical spine deformity (Fig. 6D). In the falsely predicted cases, focus was also on the subaxial spine. Interestingly, in the case where the model predicted a patient to develop no deformity based on the baseline image, a focus on a minimally degenerated subaxial cervical spine was observed (Fig. 6E). In a case where the model incorrectly predicted that the patient would develop cervical spine deformity, the subaxial spine was severely degenerated (Fig. 6F).

## 5 Discussion

In this study, we developed a deep learning model to predict the development of RA-associated cervical spine deformity based on baseline T2-weighted MRI images. The model was trained and tested using 220 MRIs of patients with no-to-minimal RA-associated changes of the craniocervical junction and/or facet joints on baseline MRI. The performance of the model was noteworthy, with an accuracy of 0.84, which indicates a correct prediction 84 % of the times. Of the 67 MRI scans in the test set, fifty-seven cases were predicted accurately. Seven scans were incorrectly predicted as positive, and three scans were incorrectly predicted as negative for the development of RA-associated cervical spine deformity, resulting in a sensitivity of 0.75 and a specificity of 0.87. A relatively stringent cut-off was applied to the confusion matrix, reflecting the model's intended use in a screening context. This choice prioritizes sensitivity and results in a lower positive predictive value and a higher negative value, aiming to minimize the risk of missing true positive cases.

In the United States, the estimated lifetime risk of developing RA is 3.6 % for women and 1.7 % for men (Crowson et al., 2011). While the number of surgeries to correct AAS and VT have steadily decreased over the past decades, the prevalence of cervical spine involvement remains present, though highly variable, and demand treatment (Lebouille-Veldman et al., 2023; Lopez-Olivo et al., 2012; Noguchi et al., 2023). Multiple studies have aimed to identify risk factors to predict development of cervical spine deformity, which would make it possible to screen patients at risk for cervical spine deformity (Momtazmanesh et al., 2022). So, the ability to detect patients at higher risk, would be of great importance. Subaxial subluxation has been associated with aggravation of cervical spine deformity, and is regarded by some as a predictive factor (Inoue et al., 2022). Other possible risk factors, and thus potential predictive factors, are long-term use of glucocorticoids, long disease duration, and the presence of anti-citrullinated protein antibodies (Baek et al., 2021; Bettaieb et al., 2023; Uchino et al., 2021). These risk factors, however, are not specific for inducing cervical deformity and cannot serve to identify patients at risk. The present algorithm to identify those patients that are at risk to develop cervical deformity has great potential to improve patient care for patients with RA. In order to use this model as a screening tool in a clinical setting, external validation is crucial.

Interestingly, the saliency maps derived from the deep learning model developed in this study suggest that degeneration of the subaxial cervical spine was important for decision making of the model and its accuracy in predicting the development of pannus and facet joint deformity at the craniocervical junction. This is congruent with the study of Inoue et al., which also identified subaxial subluxation as a risk factor for aggravation of cervical spine deformity (Inoue et al., 2022). The hypothesis rises that subaxial changes in the cervical spine could lead to deformities at the level of the craniocervical junction, because of increased laxity and movement in the subaxial spine. Further research in this subject is granted, in order to determine if it can be observed that subaxial subluxation is a true risk factor for craniocervical junction deformities later in the course of disease.

This is the first deep learning model which has been developed to predict AAS from baseline MRI. Therefore, it was not possible to compare the predictions performed by this algorithm to an existing algorithm. Also, it is not possible to compare the predictions performed by this algorithm to predictions done by neurosurgeons or radiologists, as it is not possible to predict with the naked eye from baseline scans which patients will develop cervical spine deformity at this point. It is, however, possible to diagnose cervical deformity as was done to determine the ground truth for the algorithm. For clinicians, it would be essential to know which deformities or indications of possible deformities to look for on the baseline MRI scan. Therefore, the accuracy of the opinion of the specialist would be a 50/50 chance of predicting the development of cervical spine deformity correctly.

This study has limitations. The ground truth was determined as presence or absence of cervical spine deformity on the last FU-scan of a patient. While the median FU duration between the baseline and FU scan was 7 years, it could be possible that some patients deemed as having no deformity at FU will still develop cervical spine deformity in the future. Also, multiple definitions of deformity have been used in literature in the past decades and no validated assessment has been developed. Therefore, the most often seen types of deformity were assessed for this model. It is, however, important to note that blossoming of the facet joints is an expression of biomechanical stress on the joint, and that it has also been observed in non-RA facet joints, though only sporadically (Tang et al., 2023). Also, although the positive predictive value of 0.56 suggest the model might not properly predict the presence of RA-associated cervical spine deformity at final follow-up, the accuracy, sensitivity, specificity and negative predictive value reveal the reliability of the current model, especially for screening purposes. The lower positive predictive value might lead to more false positives. Additionally, the performance of the model was highly likely limited by the number of MRIs and the unequal division of cases and non-cases (33 versus 187). Another limitation of this study is the fact that it is a single center study and uses one imaging modality, this could influence the external usefulness of the algorithm in other centers and on other imaging modalities. Furthermore, no clinical variables have been used in the model, which could influence the results of the predictions of the model. Finally, the efficacy and trustworthiness of saliency maps has not yet been determined (Arun et al., 2020, 2021).

## 6 Conclusions

In conclusion, a deep learning model was developed to predict the development of pannus and/or facet joint deformity at the craniocervical junction of patients with RA. Future research should focus on large-scale validation of this model with diverse sites and on identifying the role of the subaxial spine in the risk of deformity at the level of the craniocervical junction during the course of disease.

## Summary statement

A deep learning model was developed to predict the development of pannus and/or facet joint deformity at the craniocervical junction of patients with rheumatoid arthritis.

## Human ethics and consent to participate declarations

Not applicable.

## Clinical trial number

Not applicable.

## Ethics approval and consent to participate

The study protocol was approved under IRB number 2015P002352. The requirement for informed consent was waived.

## Consent for publication

The study protocol was approved under IRB number 2015P002352. The requirement for informed consent was waived.

## Availability of data and materials

Code will be made available upon acceptance.

## Author's contributions

All authors contributed to the conception and design of this study. ABLV and AY collected the data and did the interpretation of the data, guided by TS and CVL. AR built the deep learning model, ABLV and AY performed the analysis. ABLV, AY and AR comprised the manuscript and TS and CVL revised it critically. Supervision of this study was performed by AR, TS and CVL. All authors read and approved the final version of the manuscript before submission.

## Funding

No specific funding was received for this research.

## Declaration of competing interest

The authors declare that they have no known competing financial interests or personal relationships that could have appeared to influence the work reported in this paper.
